# Reusable Xerogel Containing Quantum Dots with High Fluorescence Retention

**DOI:** 10.3390/polym10030310

**Published:** 2018-03-13

**Authors:** Xiang-Yong Liang, Lu Wang, Zhi-Yi Chang, Li-Sheng Ding, Bang-Jing Li, Sheng Zhang

**Affiliations:** 1 Key Laboratory of Mountain Ecological Restoration and Bioresource Utilization, Chengdu Institute of Biology, Chinese Academy of Sciences, Chengdu 610041, China; liangxy1216@163.com (X.-Y.L.); luwangbest@163.com (L.W.); changzy1@cib.ac.cn (Z.-Y.C.); lsding@cib.ac.cn (L.-S.D.); 2 College of Life Sciences, University of Chinese Academy of Sciences, Beijing 100049, China; 3 State Key Laboratory of Polymer Materials Engineering, Polymer Research Institute of Sichuan University, Sichuan University, Chengdu 610065, China

**Keywords:** quantum dots, host–guest interactions, xerogel

## Abstract

Although various analytical methods have been established based on quantum dots (QDs), most were conducted in solution, which is inadequate for storage/transportation and rapid analysis. Moreover, the potential environmental problems caused by abandoned QDs cannot be ignored. In this paper, a reusable xerogel containing CdTe with strong emission is established by introducing host–guest interactions between QDs and polymer matrix. This xerogel shows high QDs loading capacity without decrease or redshift in fluorescence (the maximum of loading is 50 wt % of the final xerogel), which benefits from the steric hindrance of β-cyclodextrin (βCD) molecules. Host–guest interactions immobilize QDs firmly, resulting in the excellent fluorescence retention of the xerogel. The good detecting performance and reusability mean this xerogel could be employed as a versatile analysis platform (for quantitative and qualitative analyses). In addition, the xerogel can be self-healed by the aid of water.

## 1. Introduction

Quantum dots (QDs), mainly referring to nanocrystals of II-VI or IV-VI semiconductors, have been studied as one of the hottest research fields worldwide in recent decades due to their unique optical and electronic properties. They have the potential to bring revolutions to many technologies such as bio-imaging, (bio)sensors, solar-energy transformation, etc. [1,2,3,4]. Among the advantages of QDs, analytical chemists are most interested in the strong affinity between fluorescence property and the surface chemical conditions. Researchers have established a large trove of analytical methods based on “turn-on” or “turn-off” the fluorescence of the QDs [5,6,7,8]. However, it is difficult to find one commercial product whose operation mechanism is based on the reported methods. One of the main reasons is that most of the proposed analytical methods are based on QDs in solution, which does not benefit storage, transportation and rapid analysis, limiting their practical applications [9,10,11]. Therefore, the immobilization of QDs has attracted considerable attention in recent years.

Thus far, QD-based hydrogels have been in the center of the arena, marrying the unique photoelectrical properties of QDs with construction features of hydrogels [12,13,14]. Sol-gel method has proven that it is a powerful approach to prepare QD hydrogels. QD nanoparticles could assemble into gels spontaneously after the partial disengagement of the stabilizers [10,15,16,17]. However, the added oxidants or photochemical treatment may inactivate biomolecules, narrowing the QD applications in the field of biological detection. Even though the Eychmüller group improved the traditional sol-gel method and entrapped tyrosinase with high activity, it was still time-consuming [18]. Some other strategies based on the QDs inter-particle assembly have been developed to promote sol-gel transition, in which the elaborate design on the QDs surface was necessary [8,19,20,21,22,23,24]. A better choice to simplify the preparation procedure is constructing polymer–QDs structure. Emerging technologies, for example, layer-by-layer assembly [25], electrospun/electrospray [26,27], micropatterning [28] and inkjet/contact printing [29,30], have been employed to fabricate QDs-based sensors. Among these methods, in situ polymerization is more convenient, suitable and cost-effective for industrial manufacture, but the issues of loading capacity, stability and the size distribution of QDs should be considered primarily. Otherwise, they would lead to fluorescent properties of the final materials being uncontrollable [31,32]. Jiang et al. developed a kind of supramolecular hydrogels with QDs incorporated by host–guest interactions, in which the QDs with narrow size distribution kept their satisfactory photoluminescence, but the low QDs load (the decline of fluorescence intensity was observed when the CdS concentration exceeded 5 mg/mL) limited the practical applications of this method [33,34,35]. One might notice that almost all the above-mentioned QD immobilization proposals stopped at the hydrogel form, requiring strict storage conditions, which is unfavorable for transportation. Hydrogels could be dried at ambient atmosphere or by supercritical fluid drying system to form xerogels or aerogels, respectively [36]. Typically, aerogels dried by supercritical fluid drying system showed strong emissions (>90% of the original fluorescence intensity), while the fluorescence intensity decreased significantly (~50–60% of the original fluorescence intensity) if hydrogels had been dried at ambient atmosphere (i.e., xerogels), due to the enhanced inter-particle interactions caused by palpable volume shrinkage [9,15]. 

Another barrier to commercialize QDs-based products is the lack of environmentally friendly programs to treat the abandoned ones. Some research groups have raised the alarm that the huge risks posed by QDs should be considered seriously [37,38,39,40]. Meanwhile, the properties of each kind of QDs differs greatly from another. There is a long way to establish complete systems to dispose of the abandoned QDs.

In this paper, we developed a new strategy to prepare reusable xerogels containing QDs (cadmium telluride (CdTe), one of the typical QDs) with high fluorescence retention, which may pave the way to overcome those obstacles with following advantages: (a) the CdTe nanocrystals are immobilized in the polymer matrix via common polymerization procedure and dried at ambient atmosphere; (b) the xerogels could load a large amount of CdTe (ideally, the maximum is 50 wt % of the final xerogel) and exhibited strong emission, maintaining 85% of the QD solution fluorescence intensity; (c) the xerogels could be reused, alleviating the potential hazards caused by abandoned QDs; and (d) the presence of βCD molecules on the surface of CdTe make the xerogels potential treasure houses to be excavated (isomers, chiral distinctions, etc.) [41,42,43,44,45]. In addition, the xerogels showed the potential to act as biosensors also by entrapping corresponding enzymes.

## 2. Experimental Section

### 2.1. Materials

Cadmium chloride (CdCl_2_, anhydrous), tellurium (Te), 1-adamantanecarboxylic acid (Ad-COOH), thionyl chloride, potassium persulfate (K_2_S_2_O_8_, KPS) and *N,N,N′,N*′-tetramethylethylenediamine (TEA) were purchased from Aladdin Reagent Co., Ltd. (Shanghai, China). 2-hydroxyethyl methacrylate (HEMA), dopamine, 3,4-dimethoxyphenethylamine (DMPA) and 3-mercaptopropionic acid (MPA) were purchased from J&K Chemical Technology (Beijing, China). *N*-(hydroxymethyl)acrylamide (HMAAm) was obtained from Tokyo Chemical Industry Co., Ltd. (Tokyo, Japan) β-cyclodextrin (βCD) was purchased from Chengdu Kelong Chemical Reagent Factory (Chengdu, China). Sodium dihydrogen phosphate (NaH_2_PO_4_), thiocarbamide, sodium borohydride (NaBH_4_), and vanillin (Van) were purchased from Tianjin Kemiou Chemical Reagent Co., Ltd. (Tianjin, China) Sodium hydroxide (NaOH) and disodium phosphate dodecahydrate (Na_2_HPO_4_·12H_2_O) were purchased from Guangdong Guanghua Sci-Tech Co., Ltd. (JHD) (Shantou, China). Tyrosinase (TYR, 570 U·mg^−1^) was obtained from Beijing Solarbio Science & Technology Co., Ltd. (Beijing, China). Ultrapure water (UP water, resistivity > 18.0 MΩ·cm, 25 °C) was used in this work. All other reagents and solvents were of analytical grade and were used directly without further purification. HEMA-Ad was synthesized according to our previous works [46,47].

### 2.2. Characterization

^1^H/^13^C NMR and 2D NOESY spectra were recorded on a Bruker Avance-III 400 NMR spectrometer (Bruker, Billerica, MA, USA). Mass spectroscopy was performed using Finnigan LCQ^DECA^ mass spectrometer (Thermo Fisher Scientific, Waltham, MA, USA). FT-IR spectra of sample pellets with KBr were recorded on a Fourier transform infrared (FTIR) spectrometer Spectrum 100 (PerkinElmer, Waltham, MA, USA). SEM images of the surface of the xerogel were obtained under scanning electron microscope (JSM-7500F, JEOL, Tokyo, Japan). The UV/Vis and fluorescence spectra were detected using Varioskan Flash (Thermo). Please note that the optical properties of solutions and xerogels are different, which stemmed from the essences of different physical forms. To ensure the comparability of the collected fluorescence spectra, all controllable parameters were set as the same. The thermos-gravimetric analysis (TGA) was characterized using Thermal Analyzer EXSTAR 6000 (25–600 °C, 10 °C min^−1^, N_2_ atmosphere, Seiko Instruments Inc., Chiba, Japan).

### 2.3. Synthesis of Mono-6-thio-β-cyclodextrin (mSH-CD)

First, mono-deoxy-6-(*p*-tolylsulfonyl)-β-cyclodextrin (6-OTs-β-CD) was prepared according to the reported protocol [46] Then 6-OTs-β-CD (9.68 g, 7.51 mmol) and thiourea (11.41 g, 0.15 mol) were refluxed in 80% (*v*/*v*) methanol-water solution (400 mL) for 60 h. After the solvent was removed under vacuum, the residue was stirred in methanol for 5 h. The solid was collected by filtration and stirred in 10 wt % NaOH solution for 8 h at 50 °C. Then, the pH of the solution was adjusted to 2 with 10 wt % HCl after cooling to ambient temperature. The crud product was obtained by filtration after adding 20 mL trichloroethylene and stirred for 12 h. Finally, the crud product was stirred in methanol for 12 h and collected, and then dried under vacuum (yield: 48%). 

^1^H NMR (400 MHz, DMSO) δ = 5.86–5.57 (m, 14H, OH–2, OH–3), 4.84 (dd, *J* = 16.6, 3.4 Hz, 7H, H–1), 4.64–4.38 (m, 6H, OH–6), 3.86–3.24 (m, overlapping with HDO, H-2/3/4/5/6), 3.02–2.91 (m, 1H, H–6a which bonding with –SH), 2.81–2.69 (m, 1H, H–6b which bonding with –SH), 2.05 (dd, *J* = 10.2, 6.1 Hz, 1H, SH); MS (ESI) *m*/*z*: 1173.98 [M + Na]^+^.

### 2.4. Preparation of mSH-CD Capped CdTe (βCD-CdTe)

βCD-CdTe was synthesized according to Zhang’s study with minor changes [17]. Firstly, tellurium (40 mg, 0.3 mmol) and NaBH4 (115 mg, 3.0 mmol) were stirred in 5 mL UP water under argon atmosphere for 6 h in ice bath. Thus, colorless NaHTe solution was obtained. Secondly, MPA (52 μL, 0.6 mmol) was added in CdCl_2_ solution (110 mg, 0.6 mmol in 200 mL UP water), and then mSH-CD (345 mg, 0.3 mmol) was added. The pH was adjusted to 8 by adding NaOH solution (1 mol L^−1^). Finally, NaHTe (2.0 mL) was added to the second solution and refluxed for different times to obtain different particle size of βCD-CdTe. Argon bubbling was needed throughout the above process.

CdTe was also prepared by following the above processes but without adding mSH-CD.

### 2.5. Preparation of HEMA-Ad@βCD-CdTe Complex

The βCD-CdTe solution was concentrated to about 20 mL and stirred with HEMA-Ad (88 mg, 0.3 mmol) for 7 days in darkness. Then, the solution was filtered (microporous membrane, 0.4 μm) and dried by vacuum freeze dryer, thus HEMA-Ad@βCD-CdTe complex was obtained.

### 2.6. Preparation of the Xerogel

HMAAm (1.01 g, 10 mmol), KPS (11 mg, 0.04 mmol) and HEMA-Ad@βCD-CdTe (101 mg) were stirred in 5 mL UP water with argon bubbling for 30 min under ice bath. Then, TEA (11 μL, 0.07 mmol) was added and the mixture was transferred to a lid of 96-well plate, covered by a piece of glass. The circular grooves (diameter: 8.71 mm, depth: 0.26 mm) in the lid were used as molds to shape the HEMA-Ad@βCD-CdTe/PHMAAm hydrogel. The polymerization was conducted at room temperature for 24 h. The obtained hydrogel was rinsed with water three times and dried at room temperature for 48 h to obtain xerogels of HEMA-Ad@βCD-CdTe/PHMAAm. For recycling tests, the circular hydrogel was peeled off from the lid and dried under the same conditions.

The xerogel of HEMA-Ad@βCD-CdTe/PHMAAm loading TYR (TYR/HEMA-Ad@βCD-CdTe/PHMAAm) was prepared the same way, but displacing the solvent with PBS (pH = 6.53, 50 mM) and adding 1 mg TYR along with HMAAm.

### 2.7. Detection of Analytes

Xerogels’ fluorescence spectroscopy (λ_ex_ = 380 nm) were obtained before detecting analytes. According to the fluorescence spectroscopy, the median fluorescence intensity around the maximum emission wavelength (MFI, λ_em_ ± 5 nm) was used as the quantitative standard.

In the case of detecting Van, 5 μL of Van PBS solution (pH = 8.00, 50 mM) was added to the xerogel of HEMA-Ad-βCD-CdTe/PHMAAm. After 5 min, the median fluorescence intensity (MFI) was evaluated to calculate the concentration of the sample. For recycling test, the xerogel of HEMA-Ad-βCD-CdTe/PHMAAm was immersed in UP water for 5 min, then oscillated in dichloromethane for 10 min. Finally, the hydrogel dried at room temperature for 24 h to obtain xerogel for the next cycle of detection.

In the case of detecting dopamine, 5 μL of Van PBS solution (pH = 6.53, 50 mM) was added to the xerogel of TYR/HEMA-Ad@βCD-CdTe/PHMAAm. After incubation at 30 °C for 5 min, the MFI was measured to calculate the concentration of the sample.

### 2.8. Estimation of the Number of βCD Molecules Bound to One CdTe Particle and Calculating Inclusion Rate of HEMA-Ad@βCD-CdTe

The number of βCD molecules bound to one CdTe particle could be estimated based on TGA and TEM results.

In the range 200–400 °C, the mass loss was 42.10 wt % for βCD-CdTe, which resulted from the decomposition of MPA (*x* wt %) and βCD molecules (*y* wt %). Taking the molar ratio (*n*_MPA_:*n*_CD_ = 2:1) into account, the content of βCD in βCD-CdTe could be calculated through following formula:

(1) *x* + *y* = 42.10

(2) $$\frac{x}{M_{MPA}}=2\times \frac{y}{M_{CD}}$$

where *M*_MPA_ represents the molar mass of MPA (*M*_MPA_ = 106.14 g/mol) and *M*_CD_ represents the molar mass of βCD molecule (*M*_CD_ = 1151.05 g/mol). The calculate results are that *x* = 6.66 and *y* = 35.54, suggesting that 35.54 wt % weight loss of βCD-CdTe between 200 and 400 °C was due to βCD molecules decomposition. In other words, βCD-CdTe consists of about 0.36 g/g of βCD molecules.

The number of βCD molecules bound to one CdTe particle (*n*) could be calculated according to the following equation (assumed that 1 g of βCD-CdTe was taken):

(3) $$n=\frac{\frac{4}{3}\times \pi \times r^{3}\times \rho }{W_{CdTe}}\times \frac{W_{CD}}{M_{CD}}\times N_{A}$$

where *r* represents the radius of single CdTe nanoparticle, *r* = 2 nm (according to the TEM); *ρ* represents the density of CdTe, *ρ* = 5.85 g/cm^3^; *W*_CdTe_ represents the weight of CdTe in 1 g of βCD-CdTe particle (*W*_CdTe_ = 0.58 g); W_CD_ represents the weight of βCD in 1 g of βCD-CdTe particle (*W*_CD_ = 0.36 g); and *N*_A_ represents Avogadro constant, *N*_A_ = 6.02 × 10^23^. The calculated result is that *n* ≈ 65.

Then, the inclusion rate of HEMA-Ad@βCD-CdTe could be calculated according to the above results and the TGA result of HEMA-Ad@βCD-CdTe.

During the same temperature range (200–400 °C), the mass loss of HEMA-Ad@βCD-CdTe was 49.11 wt %, thus the content of CdTe in HEMA-Ad@βCD-CdTe is 50.89 wt %. For HEMA-Ad@βCD-CdTe and βCD-CdTe, the mass ratio among CdTe, MPA and βCD is constant. Therefore, HEMA-Ad@βCD-CdTe consists of 5.85 MPA, 31.24 βCD, 12.02 HEMA-Ad and 50.89 wt % CdTe. The inclusion rate of HEMA-Ad@βCD-CdTe (φ) could be calculated according the following equation (assuming that 1 g of HEMA-Ad@βCD-CdTe was taken):

(4) $$\varphi \;(\%)=\frac{\frac{W_{HEMA-Ad}}{M_{HEMA-Ad}}}{\frac{W_{CD}}{M_{CD}}}\times 100\%$$

where *W*_HEMA-Ad_ represents the weight of HEMA-Ad in 1 g of HEMA-Ad@βCD-CdTe, *W*_HEMA-Ad_ = 0.12 g and *M*_HEMA-Ad_ represents the molar mass of HEMA-Ad molecule, *M*_HEMA-Ad_ = 292.38 g/mol; *W*_CD_ = 0.31 g. The calculated inclusion rate of HEMA-Ad@βCD-CdTe is 152%, which is higher than 100%. The main reason is the formation of hydrogen bonds between MPA and HEMA-Ad. According to the calculated result, almost all of the βCD molecules on the CdTe surface are occupied by HEMA-Ad.

## 3. Results and Discussion

### 3.1. Preparation of the Xerogel

**Scheme 1:**
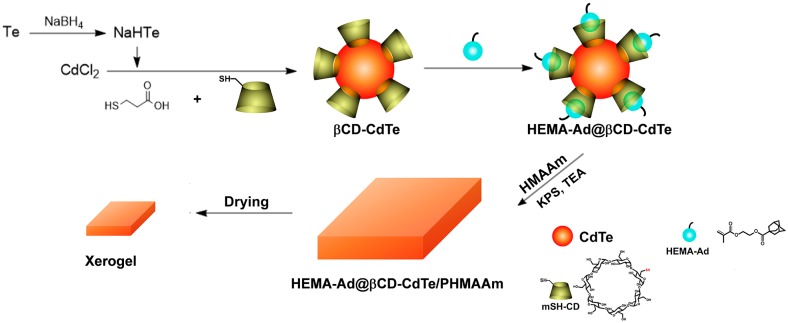
The fabrication process of HEMA-Ad@βCD-CdTe/PHMAAm xerogel.

The fabrication process of the reusable xerogel is illustrated in Scheme 1, including four steps in sequence: modifying β-cyclodextrin (βCD) molecules on the CdTe surface (termed as βCD-CdTe); introducing guest molecules (HEMA-Ad, bearing polymerization groups) via host–guest interactions (termed as HEMA-Ad@βCD-CdTe); initiating copolymerization process with KPS to obtain hydrogel (termed as HEMA-Ad@βCD-CdTe/PHMMAAm); and drying the hydrogel at ambient atmosphere to obtain the final xerogel.

Mono-6-thio-β-cyclodextrin (mSH-CD), acting as one of the stabilizers, is introduced during the synthesis of CdTe firstly [48]. The successful immobilization of βCD is confirmed by the results of Fourier transform infrared spectroscopy (FT-IR) and thermos-gravimetric analysis (TGA). In the FT-IR spectrum, βCD-CdTe shows much stronger peaks than CdTe at 1151 and 1032 cm^−1^, which stemmed from the C–O stretch and C–O–C antisymmetric vibrational modes in βCD molecules, respectively (Figure S1a, Supplementary Materials) [49]. The TGA curves show that the weight of βCD-CdTe lost 42.11% during the span of 200–400 °C, which is resulted from the decomposition of the stabilizers on the CdTe surface, including 3-mercaptopropionic acid (MPA) and mSH-CD (Figure S1b, Supplementary Materials) [50]. The amount of βCD on the surface of CdTe is about 0.31 mmol/g (about 65 βCD molecules bound to one CdTe nanoparticle), calculated from TGA results (in Experimental Section).

**Figure 1:**
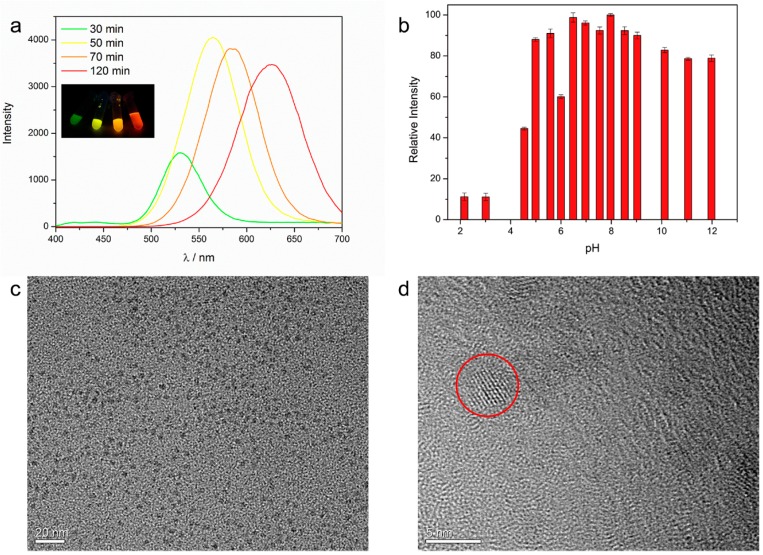
(**a**) Fluorescence spectra of βCD-CdTe at different reflux time (λ_ex_ = 380 nm). Insert is the photograph of the corresponding βCD-CdTe solutions (a small amount of the reaction solution is taken out at specific times during the reflux process) under ultraviolet lamp (λ_ex_ = 365 nm). From left to right: 30, 50, 70, 120 min. (**b**) Relative fluorescence intensity of βCD-CdTe under different pH conditions (PBS, 50 mM, βCD-CdTe: 5 mg/mL). The fluorescence intensity of βCD-CdTe at pH = 7.97 is set as 100%. (**c**) TEM (scale bar: 20 nm); and (**d**) high-resolution TEM (HRTEM, scale bar: 5 nm) images of βCD-CdTe dispersed in ultrapure water. One of the βCD-CdTe nanocrystals is marked by a red circle in the HRTEM image.

Figure 1a shows the fluorescence spectra of βCD-CdTe at different reflux time. It can be seen that the fluorescence intensity of βCD-CdTe increases significantly with the prolongation of reflux time in the first stage, and then declines slowly (Figure 1a). The increase in fluorescence intensity in the first stage is due to the crystallization of βCD-CdTe. Then, as the reflux time increases, the emission peak shifts to longer wavelength and the fluorescence intensity weakens gradually due to quantum-confined size effect [51]. Thus, the reflux process is optimized to 50 min to achieve the strongest fluorescence intensity of βCD-CdTe. The diameter of βCD-CdTe is about 4 nm, as shown in TEM images (Figure 1c,d). The fluorescence intensity of βCD-CdTe varies with different excitation wavelengths (λ_ex_). In the condition of λ_ex_ = 380 nm, βCD-CdTe exhibits the highest fluorescence intensity (Figure S2, Supplementary Materials). The fluorescence property of βCD-Cd is further investigated under different pH conditions (Figure 1b). The median fluorescence intensity (MFI) around the emission wavelength ((λ_em_ ± 5) nm) is used to quantify the fluorescence intensity of βCD-CdTe. The strongest fluorescence intensity of βCD-CdTe, which is set as 100%, is obtained under the condition of pH = 7.97. In the acidic environment, especially when the pH value is less than 4, protonation of the thiol ligands on the surface of βCD-CdTe leads to the formation of aggregates, resulting in a sharp drop-off in fluorescence intensity due to the mentioned quantum-confined size effect [52,53]. It could be observed that alkaline environment is more suitable for βCD-CdTe, as about 80% of the fluorescence intensity is still maintained even when the pH value is up to 12 (Figure 1b).

HEMA-Ad was prepared through the esterification reaction between 1-adamantanecarboxylic acid (Ad–COOH) and hydroxyethyl methacrylate (HEMA) according to our previous works [46,47]. The successful preparation of HEMA-Ad is confirmed by the results of ^1^H NMR and MS (Figure S3, Supplementary Materials).

It is well known that βCD could interact with adamantane and its derivatives to form stable inclusion complexes in various environment. The immobilized βCD molecules on the surface of βCD-CdTe could also include HEMA-Ad in their cavities to form HEMA-Ad@βCD-CdTe complex, acting as crosslinking agent in the next polymerization process. Nuclear overhauser effect spectroscopy (NOESY) is one of the most convincing means to authenticate the occurrence of inclusion between βCDs and Ad groups [46,54]. The corresponding correlation signals could be observed clearly in the NOESY spectra of HEMA-Ad@βCD-CdTe, suggesting the successful formation of assemblies (Figure S4, Supplementary Materials). Almost all of the βCD molecules on the CdTe surface are assembled with HEMA-Ad, according to the TGA results (Figure S5, Supplementary Materials, the detailed calculation process is shown in the Experimental Section). 

**Figure 2:**
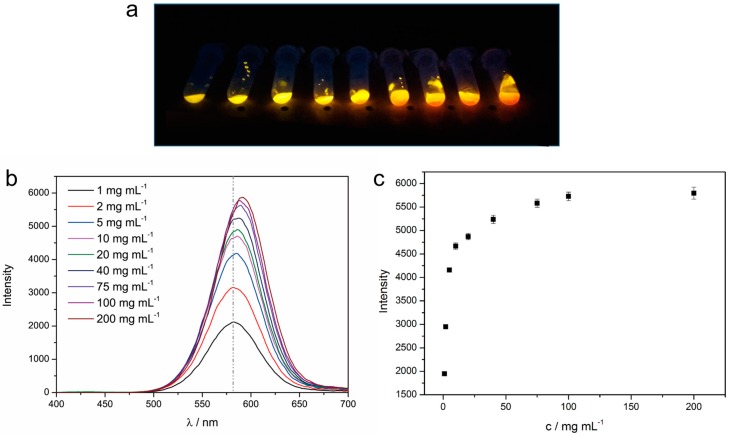
The relationship between fluorescence property and its concentration of HEMA-Ad/βCD-CdTe. (**a**) Photograph of HEMA-Ad@βCD-CdTe under ultraviolet light (λ_ex_ = 365 nm) with different concentrations (from left to right: 1/2/5/10/20/40/75/100/200 mg/mL); (**b**) fluorescence spectra of HEMA-Ad@βCD-CdTe with different concentrations (λ_ex_ = 380 nm); and (**c**) MFI scatterplot of HEMA-Ad@βCD-CdTe with different concentrations.

To optimize the dosage of HEMA-Ad@βCD-CdTe, we investigated their fluorescence properties with different concentrations. Typically, the significant decrease in the overall fluorescence intensity or redshift could be observed when the QDs concentration exceeded a certain threshold due to electronic coupling or exciton energy transfer between nanocrystals [55,56,57]. According to Jiang’s report, the threshold of concentration is about 5 mg/mL [34]. Surprisingly, in this work, no decrease in the overall fluorescence intensity and only a little redshift occurred even when the concentration of HEMA-Ad@βCD-CdTe is up to 200 mg/mL (Figure 2a,b), suggesting its excellent dispersity and stability, laying the foundation of the high loading and strong fluorescence properties of the final xerogel. We owe this prominent fluorescence retention to the introduction of βCD on the βCD-CdTe surface. βCD molecules possess cyclic structure, which could suppress the interactions between nanocrystals effectively due to steric hindrance. The solid state βCD-Cd could be re-dispersed in water easily after freeze drying. For CdTe, which is stabilized by 3-mercaptopropionic acid only, it is difficult to achieve a homogeneous dispersion solution again, since severe aggregation formed during the freeze drying (Figure S6, Supplementary Materials).

**Figure 3:**
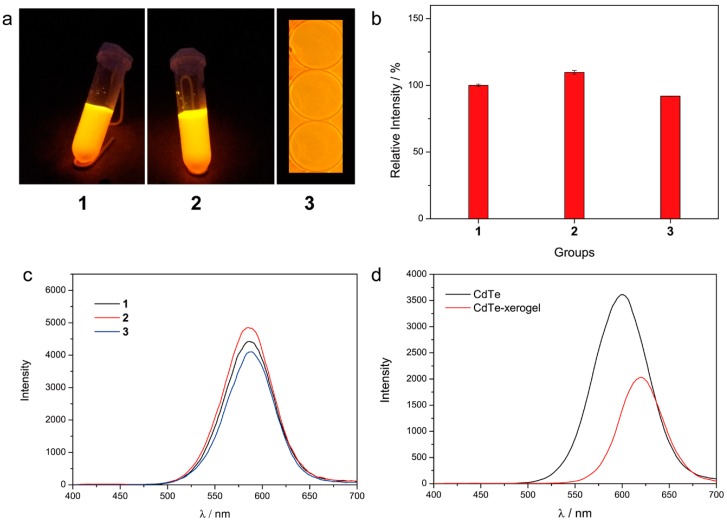
The fluorescence property of the xerogels. (**a**) photographs of βCD-CdTe (**1**, 20 mg/mL), HEMA-Ad@βCD-CdTe (**2**, 20 mg/mL) and the corresponding xerogel (**3**) under ultraviolet light (λ_ex_ = 365 nm); (**b**) the relative fluorescence intensity of **1**, **2** and **3**, in which the fluorescence intensity of **1** is set as 100%; (**c**) fluorescence spectra of **1**, **2** and **3**; and (**d**) fluorescence spectra of CdTe (20 mg/mL) and the corresponding xerogel (CdTe-xerogel). For all the fluorescence spectra, the excitation wavelength is 380 nm.

As shown in Figure 2c, in the first stage, the fluorescence intensity of HEMA-Ad@βCD-CdTe increases sharply as its concentration increases. However, the increase of fluorescence intensity slows down significantly when the concentration is higher than 20 mg/mL. Therefore, the content of HEMA-Ad@βCD-CdTe in the polymer formulation is set as 20 mg/mL. In addition, the fluorescence intensity of HEMA-Ad@βCD-CdTe is a little stronger than βCD-CdTe at the same concentration (Figure 3b,c), which may be attributed to the increased spatial distance between nanocrystals.

HEMA-Ad@βCD-CdTe/PHMAAm hydrogel was prepared by copolymerization HEMA-Ad@βCD-CdTe and HMAAm at room temperature. The final xerogel is obtained after drying the prepared hydrogel at ambient atmosphere. Predictably, shrinkage occurred during the drying process, and the surface of the xerogel is plicate (Figure S7, Supplementary Materials).

### 3.2. Fluorescence Property of the Xerogel

Ordinarily, as mentioned, QDs hydrogels dried at ambient atmosphere will result in violent decrease of fluorescence intensity, as the structure is shrunken and the density of QDs is increased (the spatial distance of adjacent nanocrystals is compressed) [55,56]. Unexpectedly, the xerogel (**3**) we prepared exhibits strong fluorescence property, whose intensity is 90% of βCD-CdTe solution (**1**), and 85% of HEMA-Ad@βCD-CdTe solution (**2**, Figure 3a,b), which is much better than reported studies (the reported fluorescence retention is about 50–60%) [9,15]. In addition, compared with the fluorescence curves of βCD-CdTe and HEMA-Ad@βCD-CdTe solution, no redshift occurs for the xerogel, suggesting that nanocrystals kept homogeneous dispersion throughout the drying process [55]. As a comparison, we also measured the fluorescence property of xerogel of CdTe/PHMAAm (CdTe-xerogel), which lacks the constraint of host–guest interactions. It could be seen that the control sample only remained 56% fluorescence intensity of the corresponding solution, along with the obvious redshift (Figure 3d), suggesting that some of the nanocrystals aggregated during the drying process. The prominent fluorescence retention of xerogel (**3**) stems from the introduction of βCD on the βCD-CdTe surface. Firstly, as we mentioned before, the βCD molecules on the particles’ surface suppressed their interactions effectively due to steric hindrance. Secondly, the quantum dot nanoparticles are immobilized in the polymer matrix through host–guest interactions, preventing the aggregations even during the drying process of the hydrogel at ambient atmosphere (the emission wavelength is almost same as the corresponding solution state, Figure 3c). 

Although our strategy is quite similar to Jiang’s, there are two very different points. Firstly, the “supramolecular cross-linker” preparation is different. Jiang employed perthiolated βCD as the sole stabilizer on the surface of CdS and the dryness process was conducted in a vacuum drier at room temperature. Aggregation of CdS was expected during the dryness process as hydrogen bonds between hydroxyl groups of βCD and the high surface energy of CdS nanocrystals. In this work, the inter-nanocrystals aggregation is suppressed effectively by employing freeze dryer. Secondly, rather than stop at the successful preparation of QDs–polymer hydrogel, we further explore the fluorescence property and versatility of the xerogel.

### 3.3. The Detecting Performance and Reusable Ability of the Xerogel

Vanillin (Van), one of the most immensely popular flavor components, having close relations to our daily life [58,59,60], is chosen as a model molecule to evaluate the detecting performance and the reusable property of the xerogels. 

**Figure 4:**
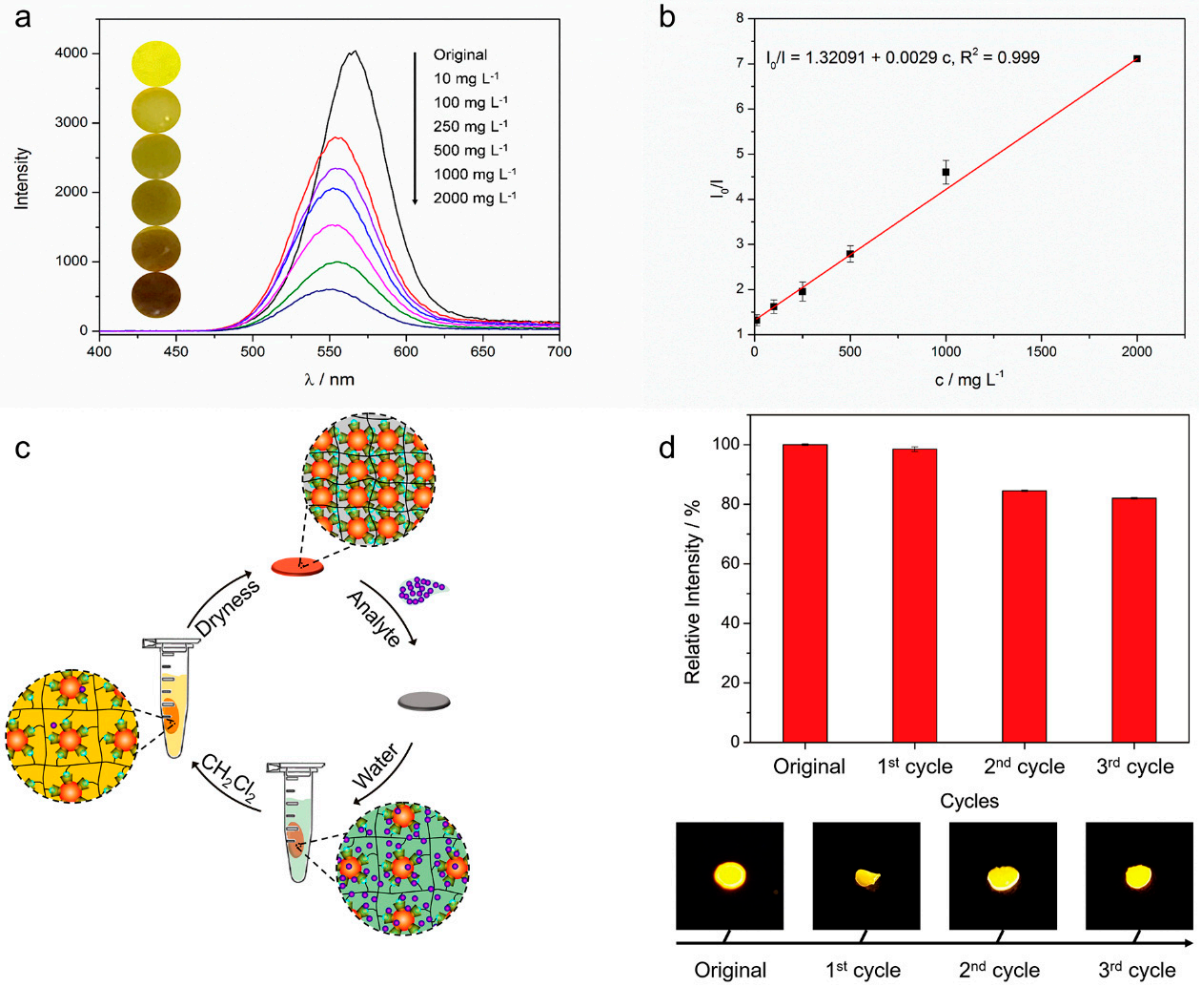
The detecting performance and reusable property of the xerogel. (**a**) Fluorescence spectra of the xerogel with different concentration Van (λ_ex_ = 380 nm). At the left is the corresponding photographs under ultraviolet light (λ_ex_ = 365 nm); (**b**) Calibration plot between *I*_0_/*I* and the concentration of Van (c, mg/L); (**c**) Schematic illustration of the reclamation process; (**d**) The relative fluorescence intensity between the original xerogel and the recycled ones. The fluorescence intensity of the original xerogel is set as 100%. Below this are photographs of the corresponding xerogels under ultraviolet light (λ_ex_ = 365 nm). For the above test samples, Van is dissolved in PBS (pH = 8.01, 50 mM).

A small amount of sample (5 μL) is required for the detection process. After adding the sample (20 mg/L Van in 50 mM PBS, pH = 8.01), the fluorescence intensity of the xerogel is monitored every 5 min. After the first 5 min, the fluorescence intensity keeps almost constant (Figure S8, Supplementary Materials). Thus, in the following experiments, the responding time between Van and the xerogel is set as 5 min. It can be seen that there is a good linear relationship between *I*_0_/*I* and the Van concentration (10–2000 mg/L): *I*_0_/*I* = 1.3209 + 0.0029 c (*R*^2^ = 0.999) (Figure 4a,b), in which *I*_0_ and *I* are the MFI of the xerogel in the absence and presence of Van, respectively, and the value of *I*_0_/*I* reflects the degree of fluorescence quenching; and c is the concentration of Van. Typically, there are two widely accepted quenching mechanisms: fluorescence resonance energy transfer (FRET) or electron transfer. As the fluorescence spectrum of xerogel does not overlap with UV–Vis spectra of Van (Figure S9, Supplementary Materials), electron transfer accounts for the fluorescence quenching phenomenon [61].

The spent xerogel could be recycled by following steps easily (Figure 4c). Firstly, the used xerogel was immersed in water for 5 min, and transformed into hydrogel due to water absorption. Secondly, the “hydrogel” was oscillated in dichloromethane for 10 min to clear Van molecules in the polymer matrix due to extraction effect. Finally, recycled xerogel was achieved after drying the “hydrogel” at room temperature for 48 h in darkness. The fluorescence of the first time recycled xerogel restored almost completely (98% of the original state, Figure 4d). Even after three cycles, the recycled xerogel kept strong fluorescence property (82% of the original state). The synergistic effect of the first two steps is necessary to achieve the extraordinary restoration of fluorescence. Soaking the utilized xerogel in water in the first step removed the remaining phosphate and relaxed the polymer chains. The host–guest interactions between βCD molecules on the CdTe surface and Ad groups linked with the polymer chains prevent quantum dots from leaking, providing a solid foundation for the fluorescence restoration. The loose polymer network structure is beneficial to clean up Van molecules owing to extraction effect in the second step.

**Figure 5:**
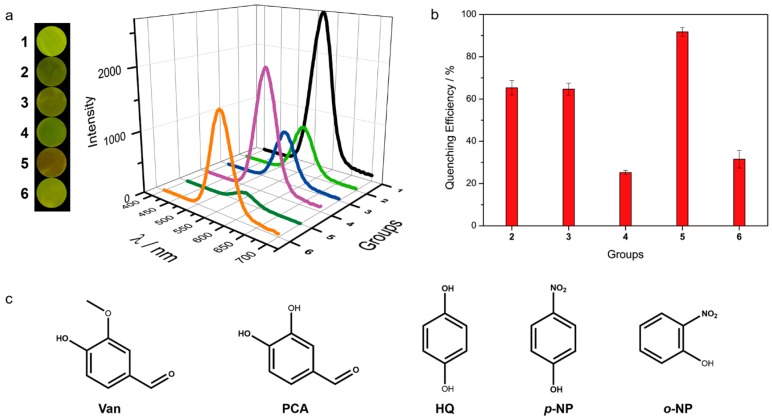
The interactions between the xerogel and various aromatic molecules. (**a**) The fluorescence spectra (λ_ex_ = 380 nm) of the xerogel after interacting with PBS (**1**, blank control, pH = 8.00), Van (**2**), PCA (**3**), HQ (**4**), *p*-NP (**5**) and *o*-NP (**6**); (**b**) The calculated quenching efficiency and (**c**) chemical structures of the detected aromatic molecules.

As shown in Figure 5a, various aromatic molecules, not only Van, could quench the fluorescence of xerogel with different degrees, including protocatechualdehyde (PCA), hydroquinone (HQ), *p*-nitrophenol (*p*-NP) and *o*-nitrophenol (*o*-NP, Figure 5c), indicating that the xerogels have great potential for acting as a versatile platform for quantitative analyze many aromatic molecules. One may notice that the quenching efficiency of *p*-NP (91.75%) is much higher than *o*-NP (31.53%) with the same concentration, suggesting that the xerogel could also be used to identify isomers (i.e., qualitative analysis) (Figure 5b) [48].

According to the mentioned experiment results, the prepared βCD-CdTe shows strong fluorescence in a wide pH range, which overlap the optimal pH range of various enzymes. By the way, polymerization process is taken place at room temperature. All of these provide an opportunity to embed enzymes in the xerogel to expand its applications. To bring this concept to reality, we attempted to encapsulate tyrosinase (TYR) in the xerogel to analyze dopamine, one of the most important neurotransmitters [62]. The ratio *I*_0_/*I* is proportional to the concentration of dopamine: *I*_0_/*I* = 1.25677 + 0.00063 c (*R*^2^ = 0.997), in which *I*_0_ and *I* are the MFI of the xerogel in the absence and presence of dopamine, respectively, *c* is the concentration of dopamine (Figure S10a,b, Supplementary Materials). The excellent linearity between *I*_0_/*I* and c results from the oxidation of dopamine to dopaquinone, which is catalyzed by TYR [63]. This could be verified through control experiments: Case 1, utilizing common xerogels (without TYR) to detect dopamine; Case 2, utilizing TYR contained xerogels to detect 3,4-dimethoxyphenethylamine (DMPA), whose chemical structure is very similar with dopamine (Figure S10d, Supplementary Materials); and Case 3, utilizing TYR contained xerogels to detect dopamine. The concentrations of all analytes are the same. As shown in Figure S10c, no significant decrease of fluorescence intensity is observed in both Case 1 and Case 2, which is quite different from Case 3. These results also indicate the specificity of the established platform to detect dopamine, certificate the fact that the xerogel is a suitable and promising candidate to fabricate various enzyme-based biosensors.

Finally, as the xerogels could be used circularly, the intrinsic self-healing property of the xerogels guarantees the long-time service. As shown in Figure S11 (Supplementary Materials), the fractured xerogel could combine again under the assistance of very little water in 5 min. The self-healing mechanism is similar to other self-healing materials containing host–guest groups [47]: the added water softens polymer network, enhancing the mobility of polymer chains; when the fractured surface contacted, the exposed βCD molecules on the CdTe surface and Ad groups linked to the polymer chain on both sides reassociate together autonomously across the interface due to host–guest interactions; at the same time, polymer chains entanglement occurred. Under the synergy of host–guest interactions and polymer chains entanglement, the fractured xerogel is restored to an entirety.

## 4. Conclusions

In summary, we fabricated a reusable xerogel containing QDs with high fluorescence retention and versatility. βCD molecules on the QDs surface are the protagonists in this strategy. Their cyclic structure suppresses interactions between nanocrystals (electronic coupling or exciton energy transfer) effectively at high concentration (up to 200 mg/mL), ensuring the high QDs loading capacity of the xerogel. Host–guest interactions between βCD molecules on the CdTe surface and Ad groups on the polymer chains immobilize QDs firmly, resulting in the excellent fluorescence retention of the xerogel, as well as self-healing capacity. The prepared xerogel could serve as not only a platform of quantitative analysis (detecting Van, for example) but also a platform of qualitative analysis (distinguishing between *p*-NP and *o*-NP, for example). The used xerogels could be recycled through three steps easily. Moreover, the xerogel could also entrap enzymes to act as biosensors.

## Supplementary Materials

The following are available online at http://www.mdpi.com/2073-4360/10/3/310/s1. Including the structure characterization of βCD-CdTe and supplementary experimental results serving for conclusions of the main text.
