# Supplementary material for: Reusable Xerogel Containing Quantum Dots with High Fluorescence Retention
Source: Polymers (Basel). 2018 Mar 13;10(3):310. doi: 10.3390/polym10030310 (PMC6414965; doi:10.3390/polym10030310)
Supplement: Supplementary file 1 [file polymers-10-00310-s001.docx]

Supplementary materials

Reusable Xerogel Containing Quantum Dots with High Fluorescence Retention

Xiang-Yong Liang ^1,2^, Lu Wang ^1,2^, Zhi-Yi Chang ^1,2^, Li-Sheng Ding ^1^, Bang-Jing Li ^1,^* and
Sheng Zhang ^3,^*

^1^ Key Laboratory of Mountain Ecological Restoration and Bioresource Utilization, Chengdu Institute of Biology, Chinese Academy of Sciences, Chengdu 610041, China; liangxy1216@163.com (X.-Y.L.); luwangbest@163.com (L.W.); changzy1@cib.ac.cn (Z.-Y.C.); lsding@cib.ac.cn (L.-S.D.)

^2^ College of Life Sciences, University of Chinese Academy of Sciences, Beijing 100049, China

^3^ State Key Laboratory of Polymer Materials Engineering, Polymer Research Institute of Sichuan University, Sichuan University, Chengdu 610065, China

***** Correspondence: libj@cib.ac.cn (B.-J.L.); zslbj@163.com (S.Z.)


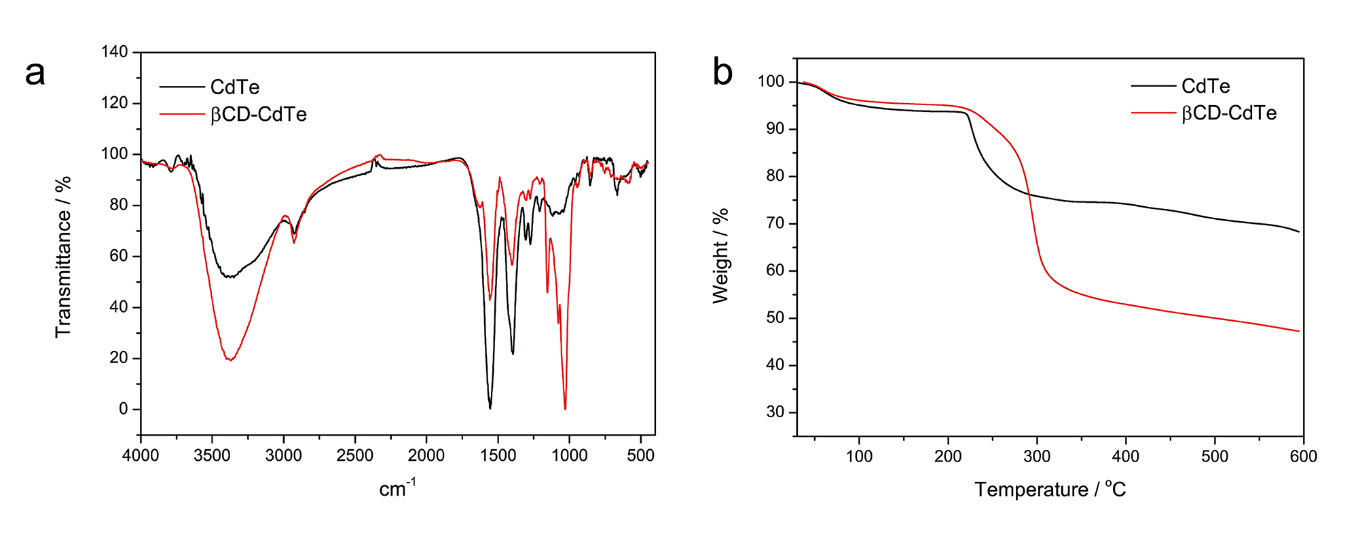


**Figure S1.** Structural characterization of βCD-CdTe. a) FT-IR spectra and b) TGA results of βCD-CdTe.


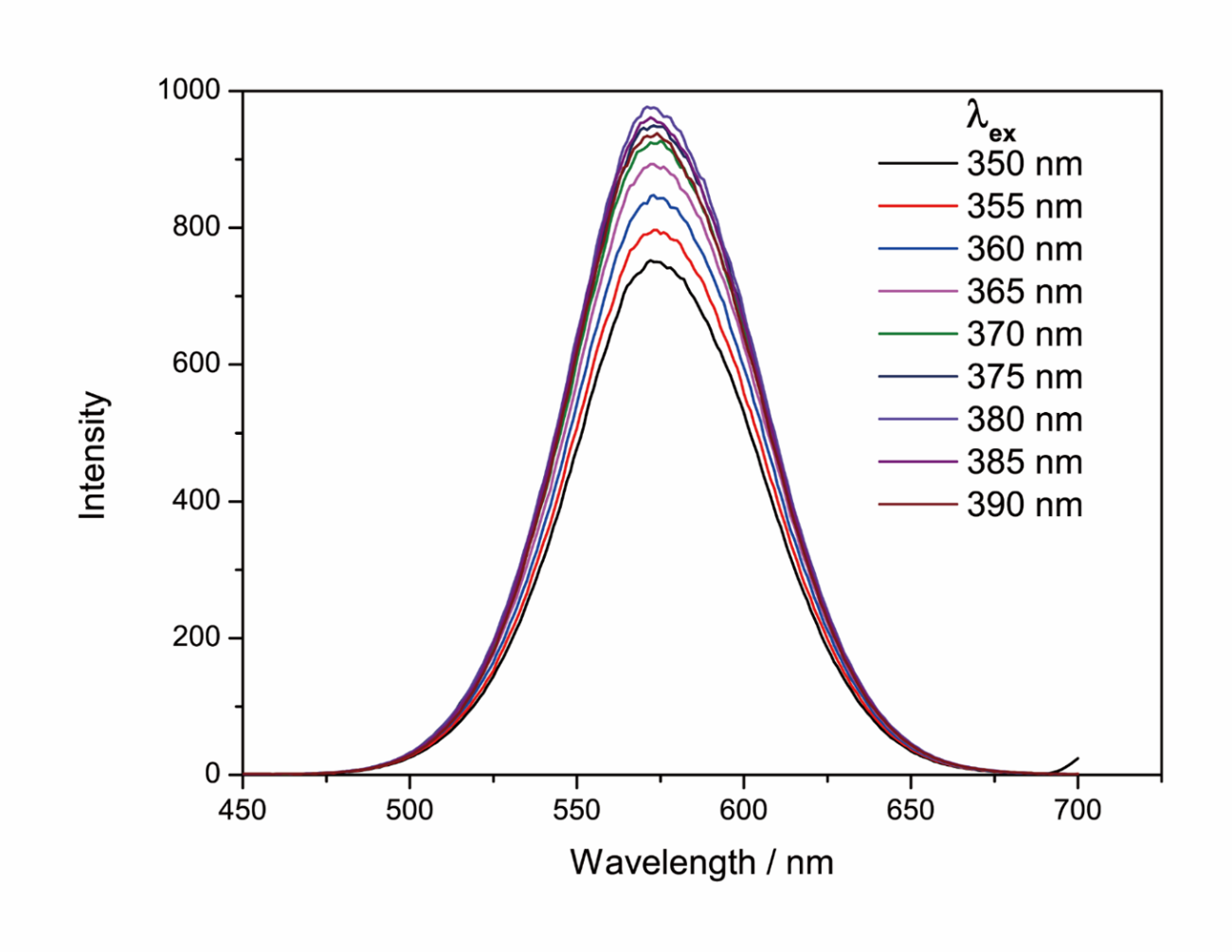


**Figure S2.** Fluorescence spectrums of βCD-CdTe solution (5 mg/mL in ultrapure water) excited by different λ_ex_.


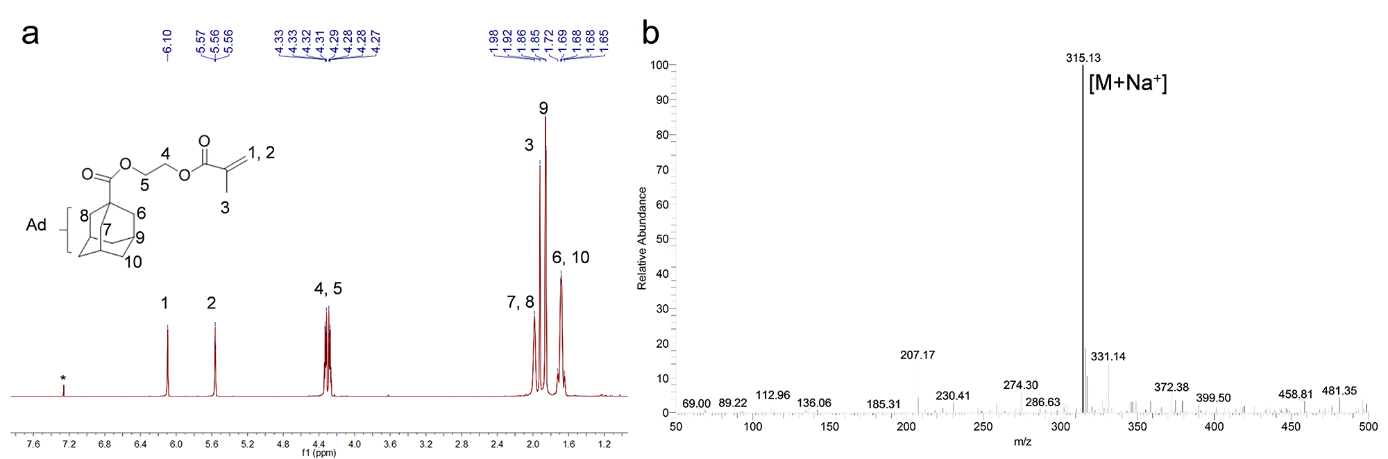


**Figure S3.** Chemical structure characterization of HEMA-Ad: ^1^H NMR (a) and MS (b) results.


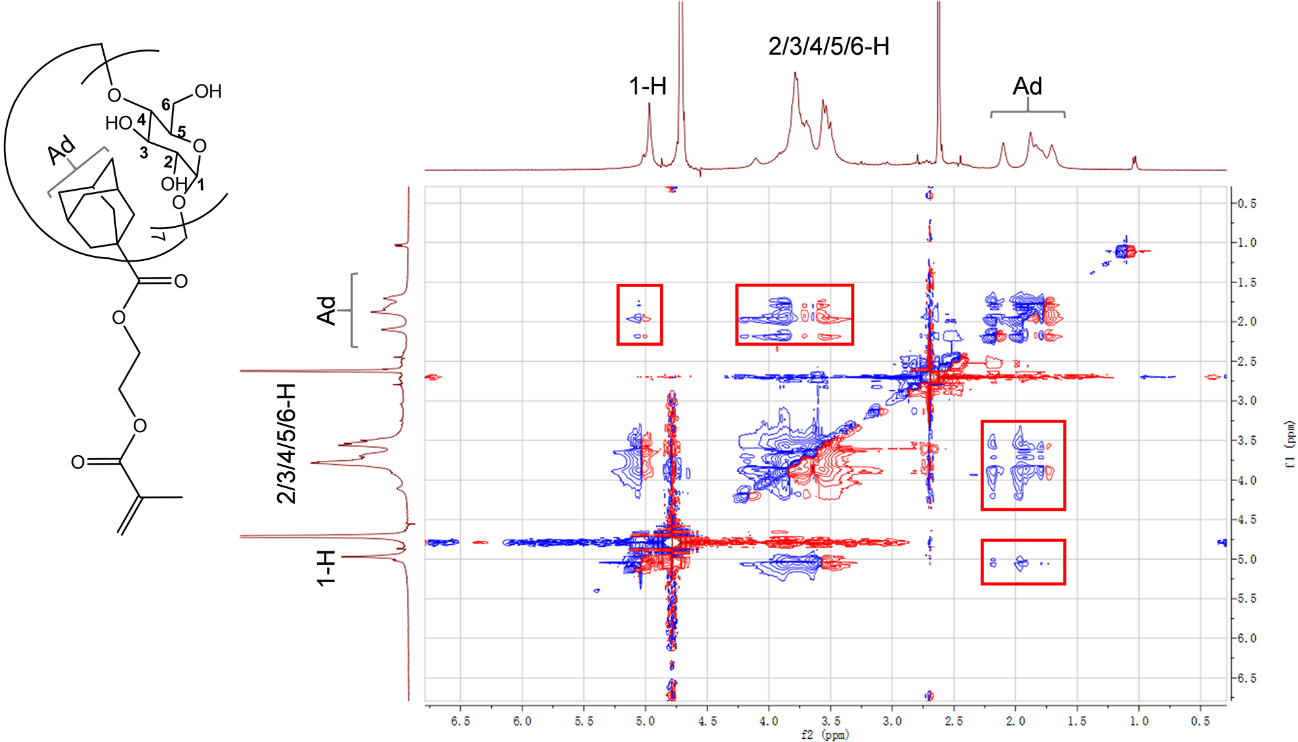


**Figure S4.** NOESY spectra of HMA-Ad@βCD-CdTe. The corresponding correlation signals are marked by red frames.


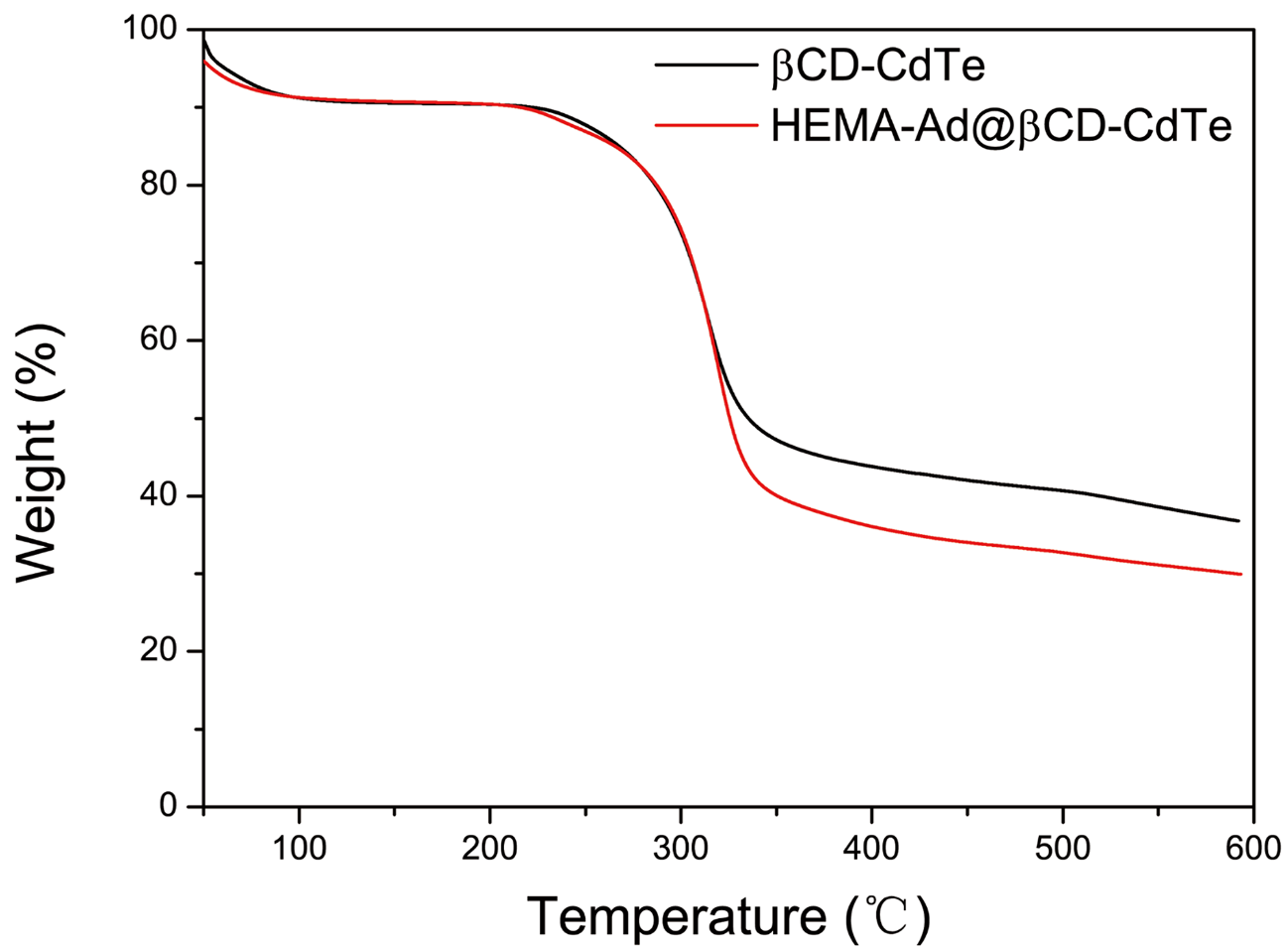


**Figure S5.** TGA results of βCD-CdTe and HEMA-Ad@βCD-CdTe (10 ℃ min^-1^ under nitrogen atmosphere).


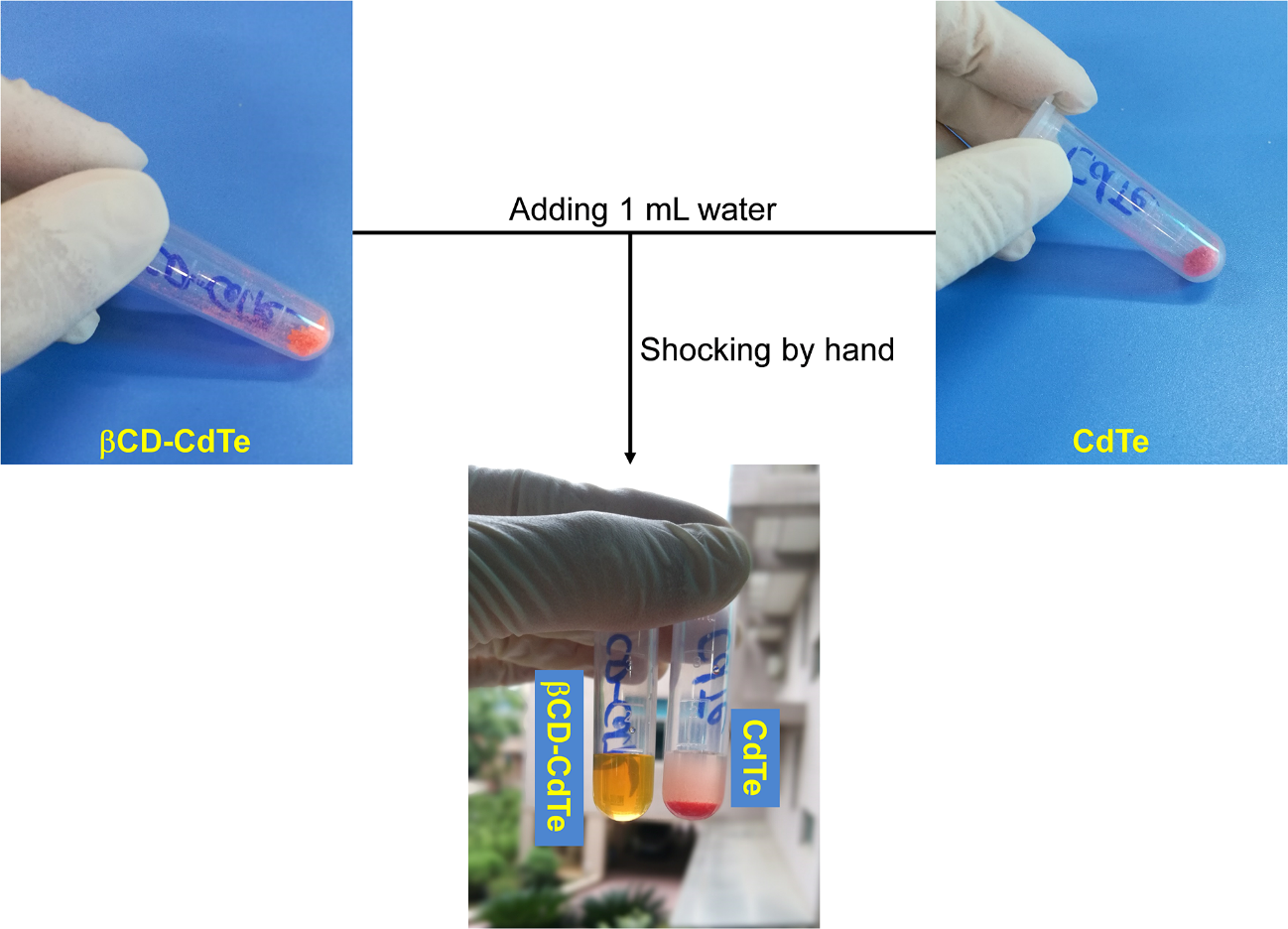


**Figure S6.** The comparison of re-disperse property between βCD-CdTe and CdTe. βCD-CdTe (5 mg) could be re-dispersed in water (1 mL) easily by shocking with hand, forming homogenous solution. While for the same amount of CdTe, most of them precipitate at the bottom after going through the same process.


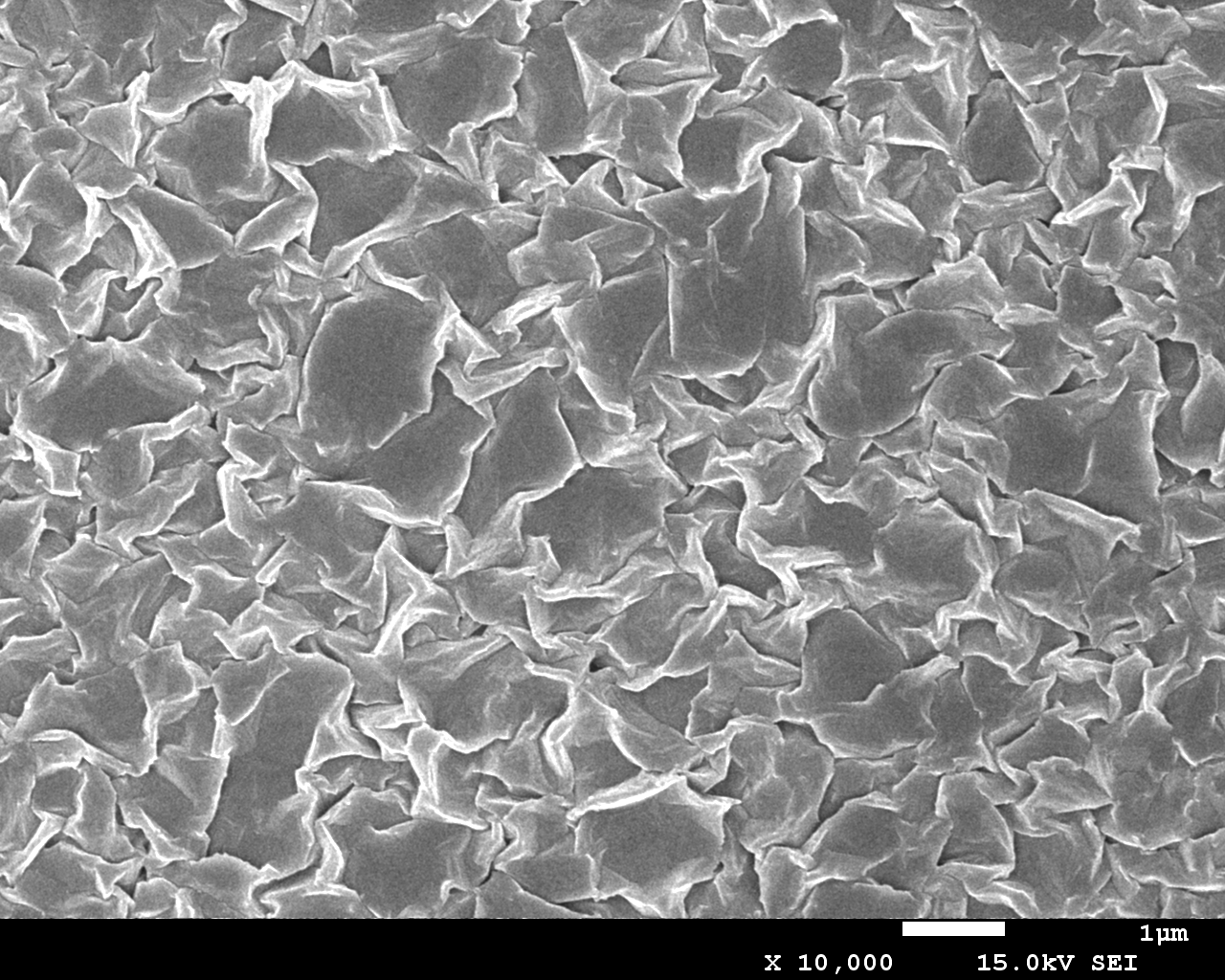


**Figure S7.** SEM image of the surface of the xerogel (scale bar: 1 μm).


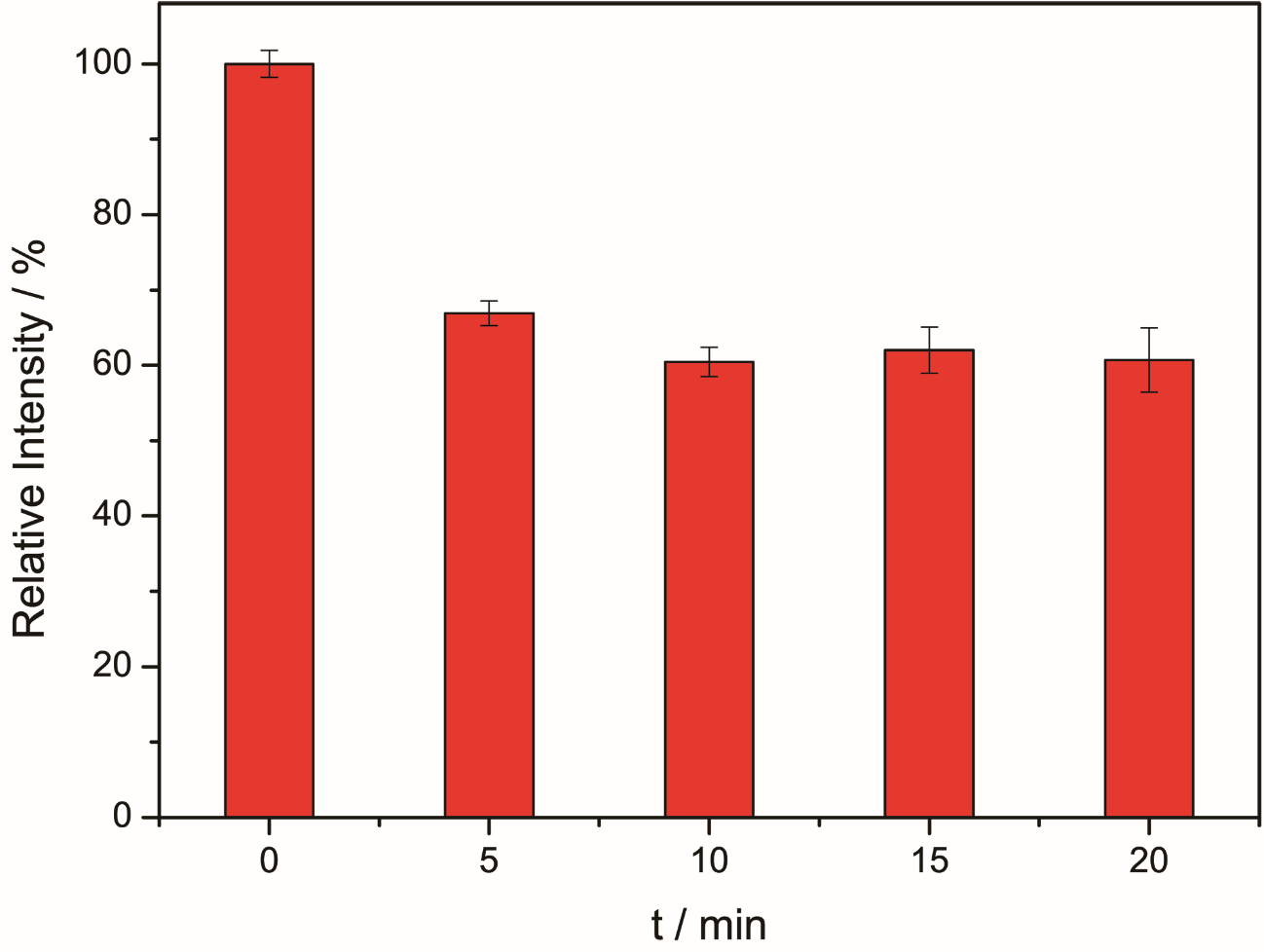


**Figure S8.** The fluorescence intensity variation verse time after adding Van sample (200 mg/L). The original fluorescence intensity of the xerogel (before adding sample) is set as 100 %.


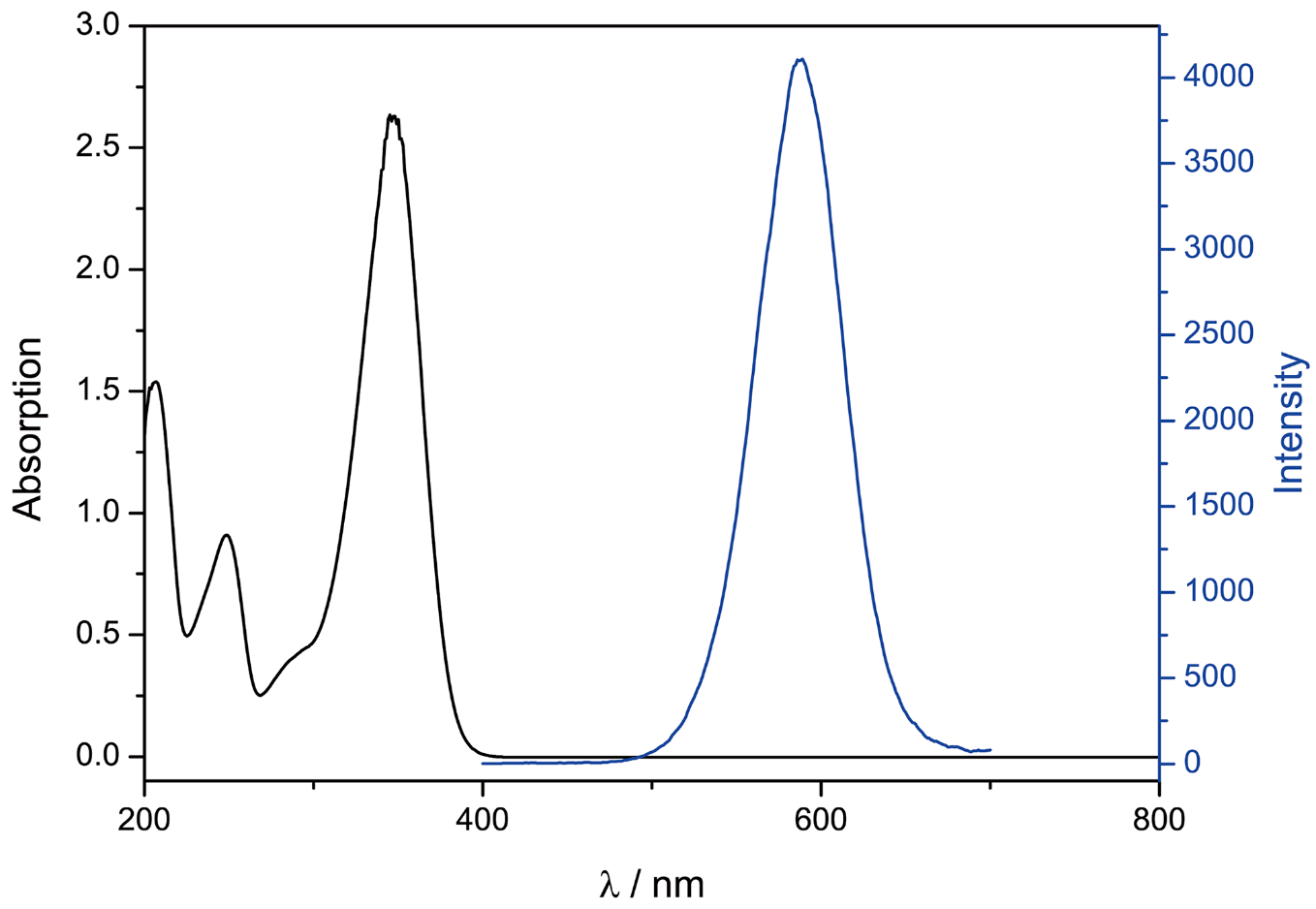


**Figure S9.** The UV-Vis absorption spectrum of Van (black line) and fluorescence spectrum of the xerogel (blue line).


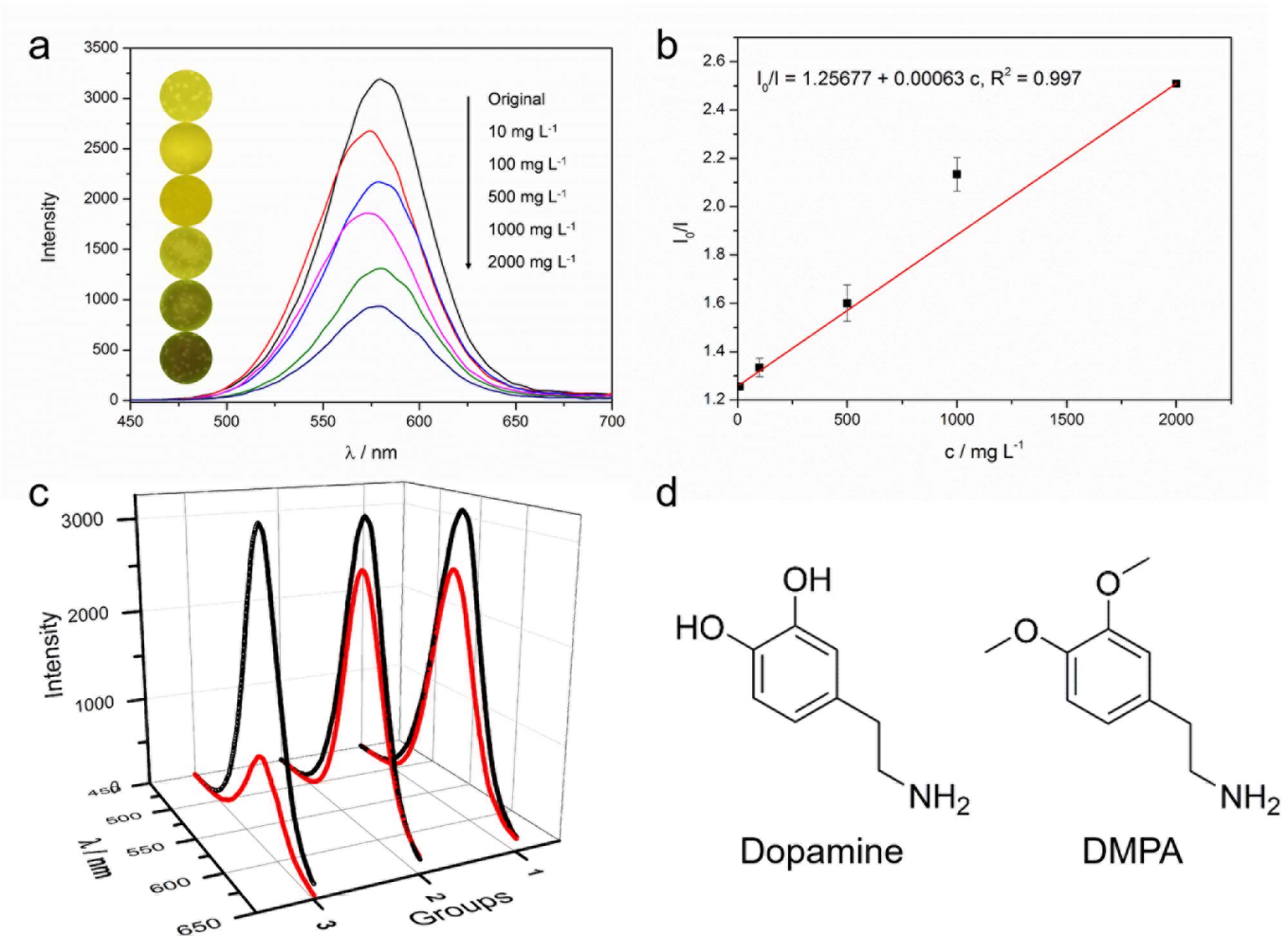


**Figure S10.** Fluorescence spectra (a) and the corresponding calibration plot (b) of the xerogel with different concentration dopamine. At the left in a) is the corresponding photographs under ultraviolet light (λ = 365 nm). Dopamine is dissolved in PBS (pH = 6.53, 50 mM). The fluorescence spectra were measured after incubation under 30 ℃ for 5 minutes. c) The fluorescence spectra of the xerogels before (black lines) and after adding analysts (red lines), respectively. **Case 1**: utilizing common xerogels (without TYR) to test dopamine. **Case 2**: utilizing TYR contained xerogels to test DMPA. **Case 3**: utilizing TYR contained xerogels to test dopamine. The concentrations of all analytes are 10.56 mM in PBS (pH = 6.07, 50 mM). All of the detection processes are consistent with detecting dopamine. d) Chemical structures of dopamine and DMPA, respectively.


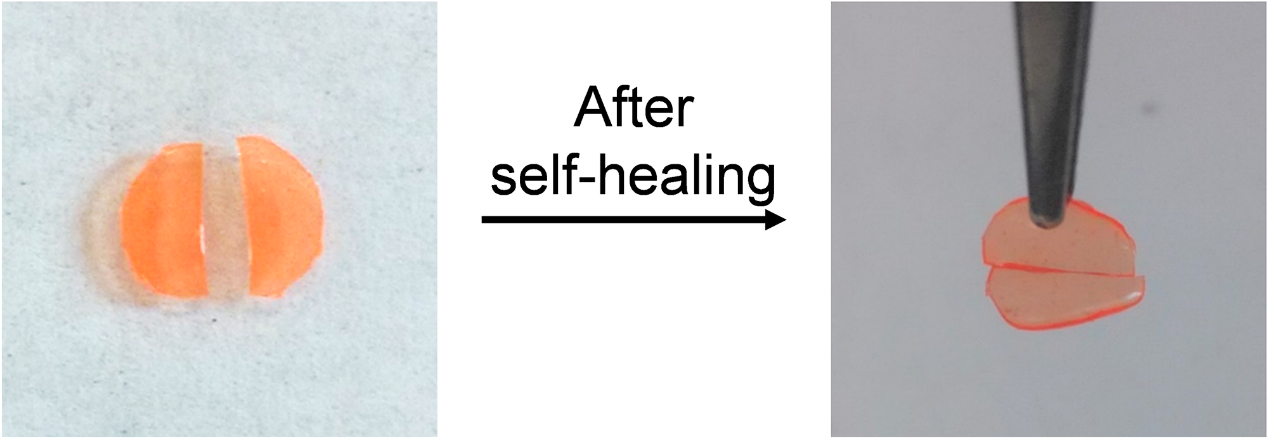


**Figure S11.** The self-healing process of the fractured xerogel. The xerogel is cut in half by a scalpel (left). Adding 1 μL water at the fractured surfaces and putting the fractured surfaces together for 5 minutes. Finally, the fractured xerogel rejoined into an entirety spontaneously (right).
